# Regulation of PINX1 expression ameliorates lipopolysaccharide-induced lung injury and alleviates cell senescence during the convalescent phase through affecting the telomerase activity

**DOI:** 10.18632/aging.202779

**Published:** 2021-03-26

**Authors:** Shujing Li, Bin Jiang, Haiyang Yu, Dongqing Song

**Affiliations:** 1 Rehabilitation Medicine Department, Qingdao Hospital of Traditional Chinese Medicine (Hiser Hospital), Qingdao 266033, Shandong Province, China; 2 Intensive Care Unit, Qingdao Hospital of Traditional Chinese Medicine (Hiser Hospital), Qingdao 266033, Shandong Province, China

**Keywords:** acute lung injury, PINX1, telomerase reverse transcriptase, cell senescence, inflammation

## Abstract

PIN2/TERF1-interacting telomerase inhibitor 1 (PINX1) is necessary for telomerase reverse transcriptase (TERT) elements to bind at telomeres and non-telomere sites. We aimed to investigate the role of PINX1 and TERT in lipopolysaccharide (LPS)-induced lung injury during acute stage and convalescent phase. Lung injury rat model was induced, and the expression of PINX1 and TERT in serum and lung tissues was examined using RT-qPCR on day 0 (D0), D3, and D14, respectively. The pathologic changes of lung tissues on D3 and D14 were detected using hematoxylin and eosin staining after TERT overexpression, PINX1 overexpression, or PINX1 silencing in lung injury rats. Results revealed that TERT was persistently reduced on D3 and D14, while PINX1 was decreased on D3 but increased on D14. TERT overexpression and PINX1 silencing led to the most serious lung damage, the highest levels of inflammatory factors and apoptosis on D3, while the best recovery was observed on D14. Simultaneously, PINX1 overexpression presented the opposite effects at acute stage and convalescent phase. Co-immunoprecipitation (co-IP) assay verified the connection between PINX1 and TERT. Taken together, these findings demonstrated that regulation of PINX1 expression ameliorates lung injury and alleviates cell senescence during the convalescent phase through affecting the telomerase activity.

## INTRODUCTION

Sepsis is a systemic inflammatory response syndrome caused by infection, which increases the incidence and mortality of several organ injuries [1]. Acute lung injury (ALI) is one of the most common complications of sepsis resulting from pulmonary susceptibility [2]. ALI, a critical syndrome with worryingly high morbidity and mortality in intensive care patients, leads to edema, hypoxemia, and even acute respiratory distress syndrome (ARDS) [3, 4]. Uncontrolled acute inflammatory response and extensive apoptosis of pulmonary alveolar type II epithelial cells are considered to be closely related to development of ALI [5, 6]. Although enormous efforts and great progress have been made in recent years, there is still no effective method for ALI treatment [7]. Therefore, there is high demand for a better understanding of ALI pathogenesis to identify effective therapies for ALI.

Telomeres and the activity of telomerase are closely associated with cell survival and senescence. As a special structure of the ends of chromosomes, telomeres play vital roles in maintaining and controlling the complete structure of chromosomes and the senescence and carcinogenesis of cells [8]. A large amount of evidence shows that inflammation and oxidative stress are the factors affecting cell aging, among which the growth rate of telomere length shortening is considered to be one of the important factors [9, 10]. Some scholars have found that acute inflammation often presents a series of aging manifestations after being cured, such as decreased organ function, abnormal metabolic level, and the early emergence of some senile diseases, which are often accompanied by abnormal shortening of telomere length [11].

PIN2/TERF1-interacting telomerase inhibitor 1 (PINX1), an inhibitor of telomerase activity, is necessary for telomerase reverse transcriptase (TERT) elements to bind at telomeres and non-telomere sites [12]. It has been reported that TERT could attenuate lung fibrosis via protecting alveolar epithelial cells against senescence [13]. PINX1 is considered as a key component of TERT/telomerase homeostasis through its ability to bind to TERT and inhibit TERT activity [12]. TERT plays an important role in regulating the expression of telomerase activity by epigenetic regulations and telomere position effect. However, it is noteworthy that inhibition of TERT reduced the level of tumor necrosis factor alpha (TNF-α) by inactivation of nuclear factor-kappa B (NF-κB) signaling [12]. Interestingly, low doses of PINX1 promoted NF-κB expression while high doses produced the opposite inhibitory effect. Further studies have found that PINX1 and p65 have a co-expression relationship, and the biphasic action of PINX1 is related to the different binding domains and corresponding functions of its c-terminal and n-terminal [12].

The present study aimed to investigate the expression levels of PINX1 and TERT in LPS-induced lung injury model from the acute stage to the convalescent phase. Furthermore, by regulating the level of PINX1, the changes in telomerase activity and the degree of inflammation and apoptosis at various stages were observed to reveal the regulatory effect of PINX1 on ALI and its underlying mechanism.

## RESULTS

### The changes in TERT and PINX1 expression during the acute stage and convalescent phase of lung injury in rats induced by LPS

**Figure 1:**
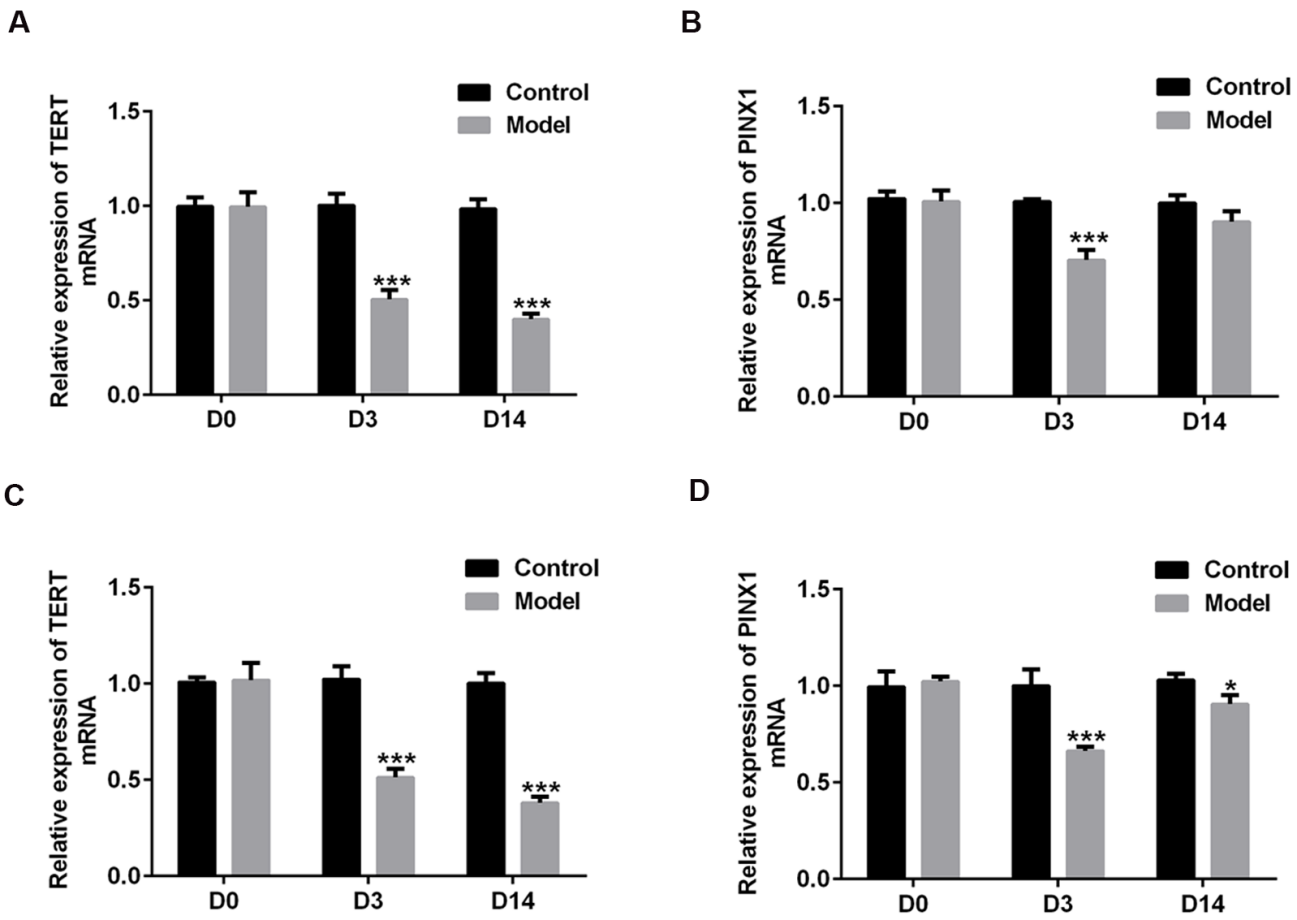
**The changes in TERT and PINX1 expression during the acute stage and convalescent phase of lung injury in rats induced by LPS.** The levels of (**A**) TERT and (**B**) PINX1 in serum and (**C**) TERT and (**D**) PINX1 levels in lung tissues were determined using RT-qPCR. ^*^P<0.05, ^***^P<0.001 vs. Control. TERT, telomerase reverse transcriptase; LPS, lipopolysaccharide.

To explore the roles of TERT and PINX1 in the acute stage and convalescent phase of lung injury rats induced by LPS, the expression of both TERT and PINX1 in serum and lung tissues of rats was detected using RT-qPCR on D3 and D14. As shown in Figure 1A, 1B, TERT level in serum was persistently reduced on D3 and D14 compared with the control group, while PINX1 expression was decreased on D3, but increased on D14 compared to D3. Both TERT and PINX1 expression in lung tissues exhibited the same variation trends as that in serum (Figure 1C, 1D). These data revealed that the levels of TERT and PINX1 presented different changes from the acute stage to convalescent phase of rats with lung injury.

### TERT overexpression or PINX1 silencing aggravated the changes of lung tissue pathobiology on D3 while PINX1 overexpression alleviated it in LPS-induced lung injury rats

**Figure 2:**
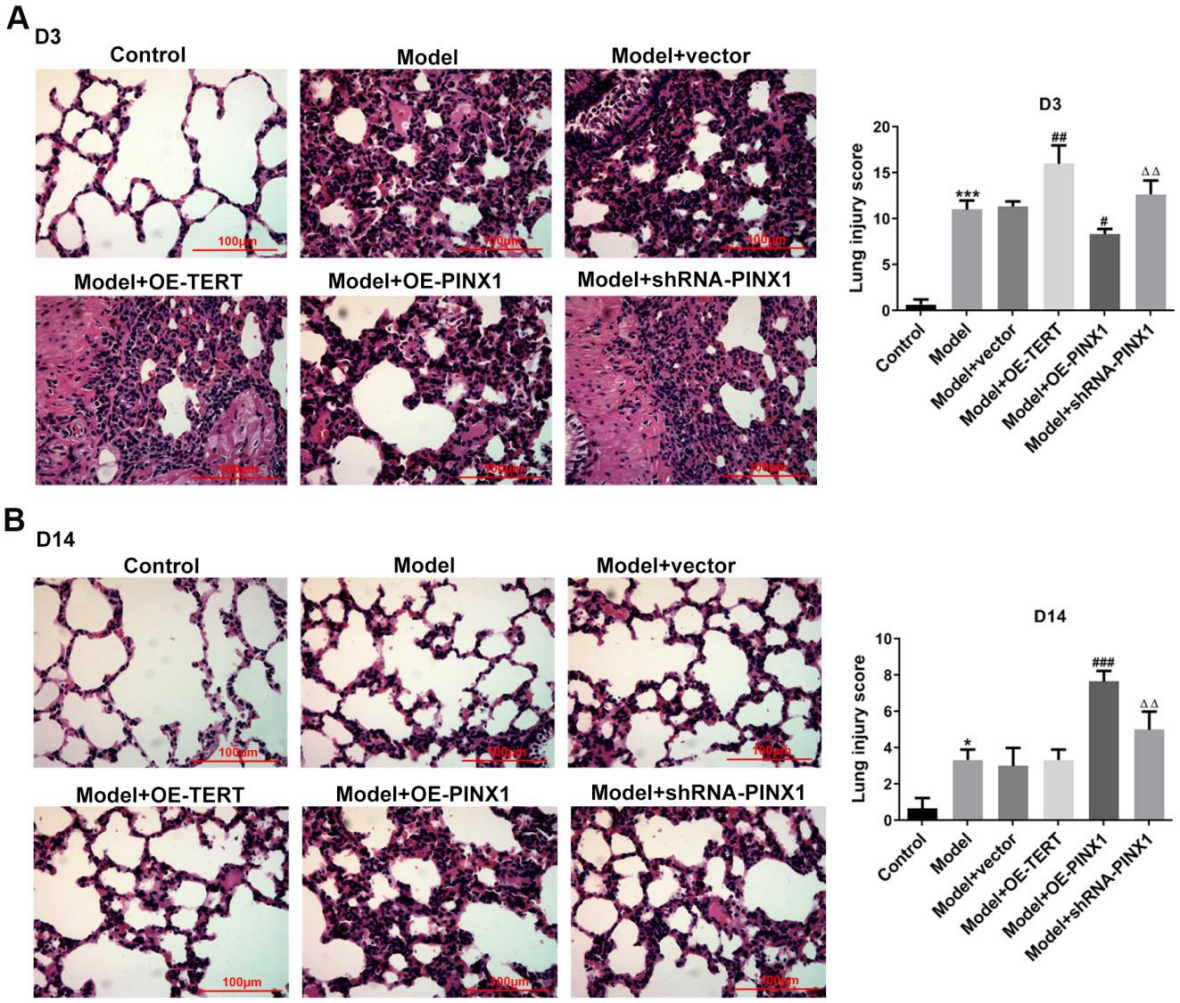
**TERT overexpression, PINX1 silencing, and PINX1 overexpression presented different effects on pathological changes of lung tissue on D3 and D14 in LPS-induced lung injury rats.** The histopathological changes in lung tissues on (**A**) D3 and (**B**) D14 were evaluated using H&E staining. ^*^P<0.05, ^***^P<0.001 vs. Control; ^#^P<0.05, ^##^P<0.01, ^###^P<0.001 vs. Model+vector; ^ΔΔ^P<0.01 vs. Model+OE-PINX1.

To investigate the regulatory effects of TERT and PINX1 in lung injury rats induced by LPS, the pathological changes of lung tissue sections on D3 were determined using H&E staining. It is observable in Figure 2A that rats in the control group displayed normal pulmonary alveolar structure, while animals in the model group showed destructive alveolar structure, thickened alveolar septal walls, visible vascular congestion, and inflammatory cell infiltration. Remarkably, TERT overexpression or PINX1 silencing further exacerbated the pathological changes of lung tissue whilst PINX1 overexpression relieved them. These results implied that TERT and PINX1 play different roles in the acute phase of LPS-induced lung injury in rats.

### TERT overexpression or PINX1 silencing promoted the recovery of lung tissues on D14, whereas PINX1 overexpression led to a slower recovery of lung injury

Subsequently, the pathological changes of lung tissues on D14 were measured to analyze the effects of TERT and PINX1 on the convalescent phase of lung injury rats induced by LPS. Results in Figure 2B indicated that rats in the model group displayed significant improvement in lung injury relative to the control group. Rats with TERT overexpression or PINX1 silencing presented notable recovery from lung tissue injury on D14 compared with lung injury on D3. By contrast, the most severe lung tissue damage was seen on D14 when PINX1 was overexpressed. Collectively, these findings suggested that TERT overexpression or PINX1 silencing contributed to the recovery of lung tissue changes in the convalescent phase of lung injury, whereas PINX1 overexpression resulted in a slower recovery of lung injury.

### TERT overexpression or PINX1 silencing exacerbated inflammatory response on D3 while promoted the recovery of inflammatory injury on D14, whereas PINX1 overexpression presented the opposite effects in LPS-induced lung injury rats

**Figure 3:**
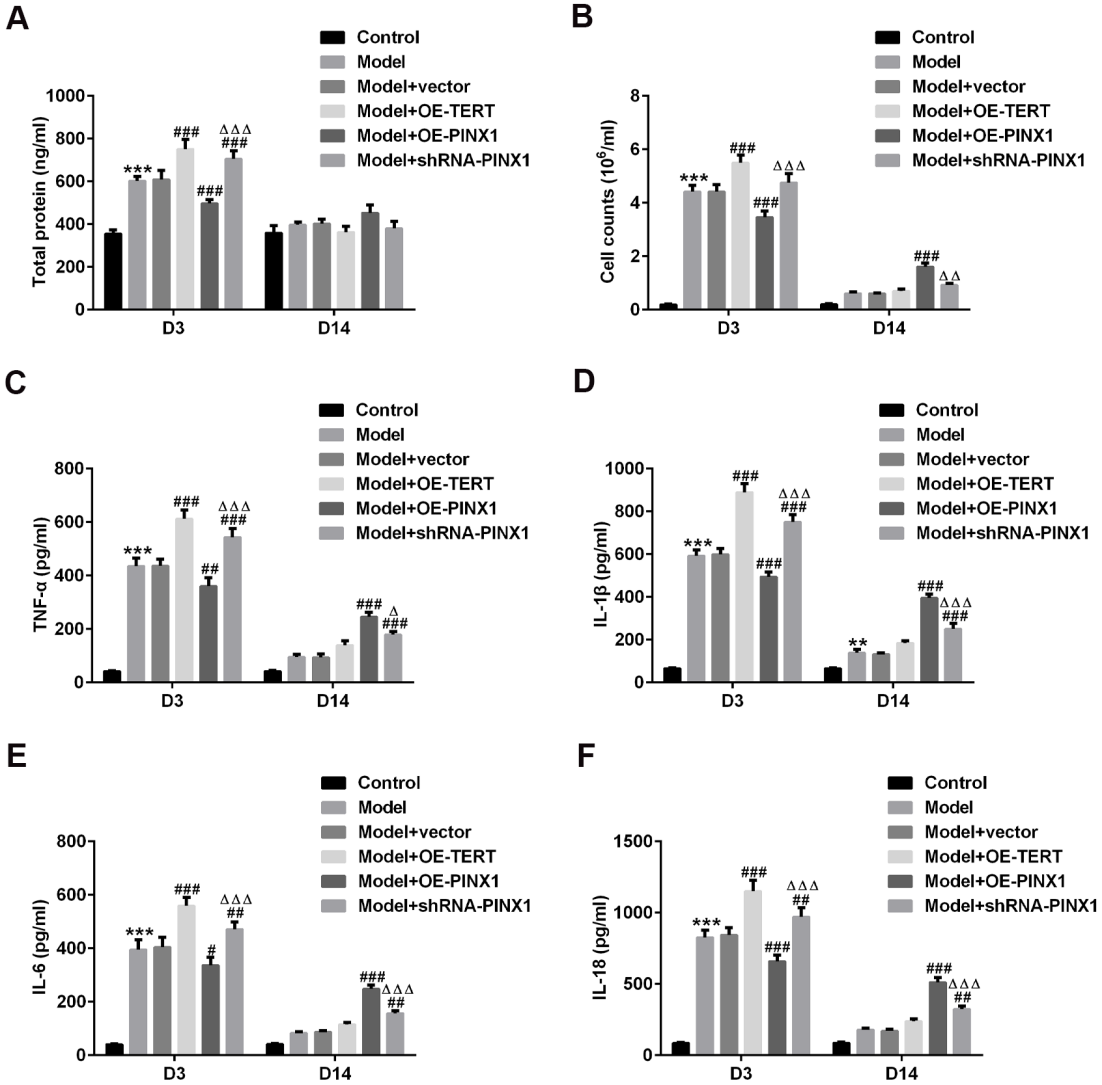
**TERT overexpression or PINX1 silencing exacerbated inflammatory response at D3 while promoted the recovery of inflammatory injury on D14, whereas PINX1 overexpression presented the opposite effects in LPS-induced lung injury rats.** (**A**) A bicinchoninic acid (BCA) Protein Assay Kit was used to test the protein concentration in BALF. (**B**) The contents of cells in BALF were counted by an automatic cell counter. The levels of (**C**) TNF-α, (**D**) IL-1β, (**E**) IL-6, and (**F**) IL-18 were assessed using ELISA kits. ^**^P<0.01, ^***^P<0.001 vs. Control; ^#^P<0.05, ^##^P<0.01, ^###^P<0.001 vs. Model+vector; ^Δ^P<0.05, ^ΔΔ^P<0.01, ^ΔΔΔ^P<0.001 vs. Model+OE-PINX1.

The total proteins in BALF of rats in each group were examined using commercial kits. As exhibited in Figure 3A, 3B, the content of total proteins and the inflammatory cell count on D3 in the model group were significantly increased, which were further enhanced after TERT overexpression or PINX1 silencing. Conversely, PINX1 upregulation dramatically decreased them as compared to the model+vector group. Subsequently, the levels of above-mentioned indicators in BALF on D14 after the model establishment were tested. As could be observed in Figure 3A, 3B, the total protein and the inflammatory cell count were reduced overall compared with those on D3. Differently, the total protein and the number of inflammatory cells in the TERT overexpression group and the PINX1 silencing group were significantly reduced on D14, whereas PINX1 overexpression presented the highest levels of protein content and inflammatory cell count. Consistently, results of the levels of inflammatory factors including TNF-α, IL-1β, IL-6, and IL-18 in different groups shown the same variation trends as the total protein and the number of inflammatory cells (Figure 3C–3F). To sum up, above data demonstrated that TERT overexpression or PINX1 silencing exacerbated inflammatory response in the acute phase while promoted the recovery of inflammatory injury in the convalescent phase, whereas PINX1 overexpression presented the opposite effects.

### TERT overexpression or PINX1 silencing aggravated apoptosis on D3 while promoted the recovery of apoptosis on D14, whereas PINX1 overexpression presented the opposite effects

**Figure 4:**
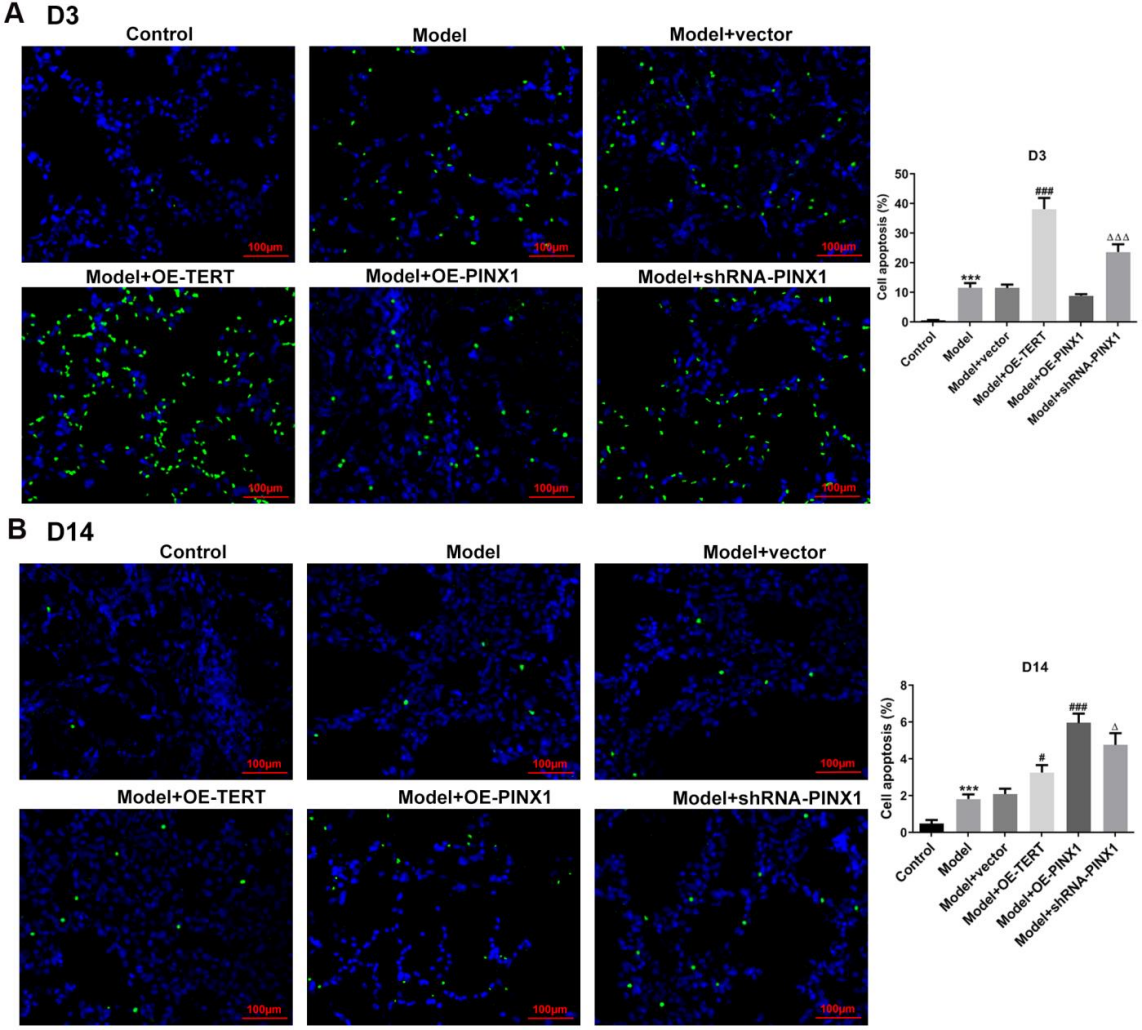
**TERT overexpression or PINX1 silencing aggravated apoptosis on D3 while promoted the recovery of apoptosis on D14, whereas PINX1 overexpression presented the opposite effects.** Cell apoptosis on (**A**) D3 and (**B**) D14 was detected using TUNEL staining. ^***^P<0.001 vs. Control; ^#^P<0.05, ^###^P<0.001 vs. Model+vector; ^Δ^P<0.05, ^ΔΔΔ^P<0.001 vs. Model+OE-PINX1.

**Figure 5:**
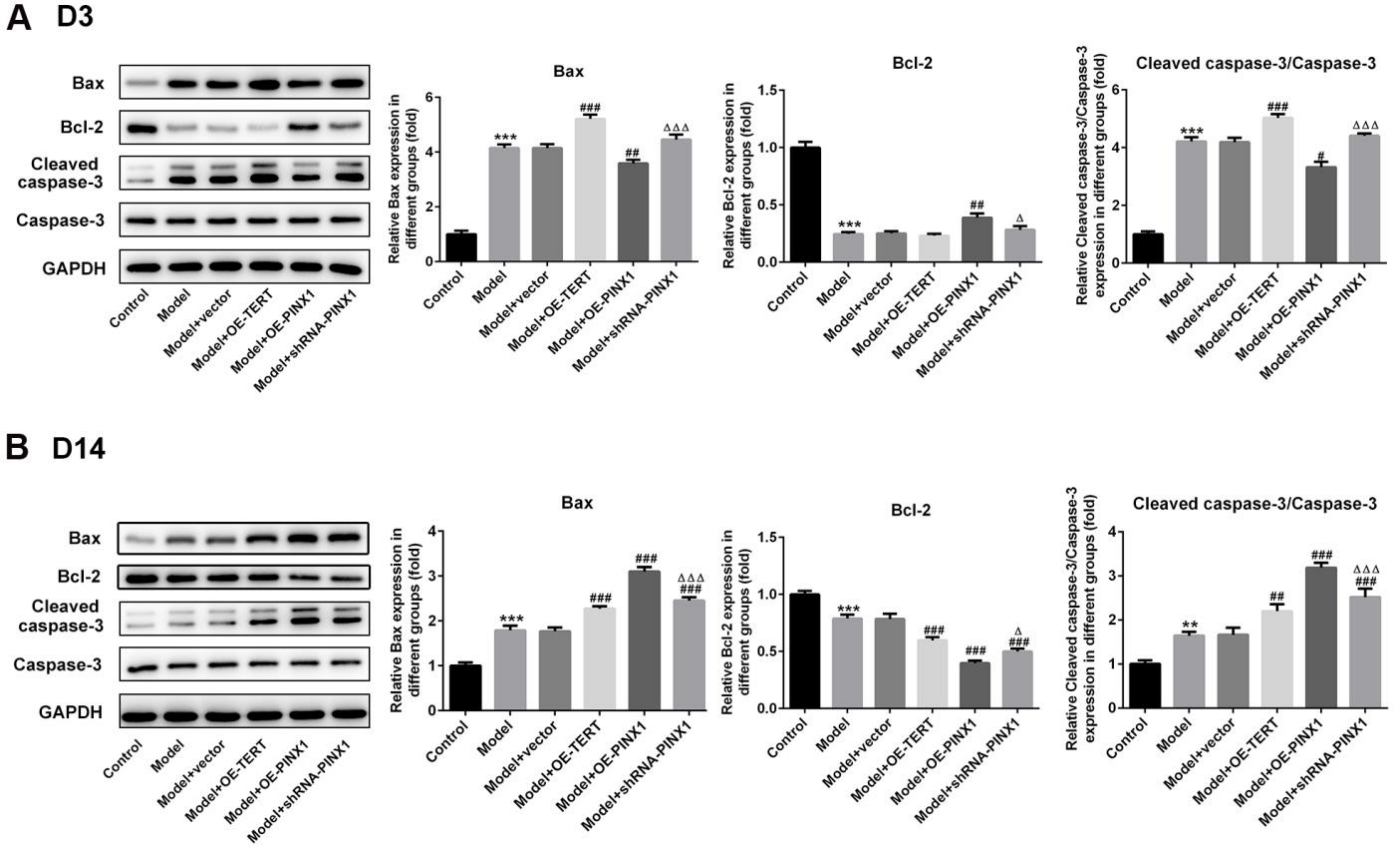
**TERT overexpression, PINX1 silencing or PINX1 overexpression presented different effects on the expression of apoptosis-related proteins.** The expression of apoptosis-related proteins on (**A**) D3 and (**B**) D 14 was detected using western blot analysis. ^**^P<0.01, ^***^P<0.001 vs. Control; ^#^P<0.05, ^##^P<0.01, ^###^P<0.001 vs. Model+vector; ^Δ^P<0.05, ^ΔΔΔ^P<0.001 vs. Model+OE-PINX1.

TUNEL staining was employed to assess apoptosis on D3 and D14. As displayed in Figure 4A, cell apoptosis was dramatically enhanced in the model group relative to the control group. After TERT upregulation or PINX1 downregulation, apoptosis aggravated, whilst PINX1 overexpression showed the lowest level of apoptosis on D3. However, cell apoptosis was significantly decreased on D14, but different groups exhibited different degrees (Figure 4B). The TERT overexpression group and the PINX1 silencing group exhibited low levels of apoptosis, while the PINX1 overexpression group showed the most obvious apoptosis on D14. Concurrently, the expression of apoptosis-related proteins was determined using western blot analysis. Results in Figure 5A indicated that the expression of Bax and cleaved caspase-3 was notably upregulated accompanied by obviously downregulated Bcl-2 expression on D3 after TERT overexpression or PINX1 silencing, compared with the model+vector group. By contrast, PINX1 overexpression dramatically decreased the levels of Bax and cleaved caspase-3 while increased that of Bcl-2. Moreover, on D14, TERT overexpression or PINX1 silencing presented significant decrease in Bax and cleaved caspase-3 expression coupled with an obvious increase in Bcl-2 expression (Figure 5B). Meanwhile, PINX1 overexpression exhibited the opposite effects. These findings provided a clue that TERT overexpression or PINX1 silencing aggravated apoptosis on D3 while promoted the recovery of apoptosis on D14, whereas PINX1 overexpression presented the opposite effects.

### PINX1 regulated the telomerase activity via directly interacting with TERT

**Figure 6:**
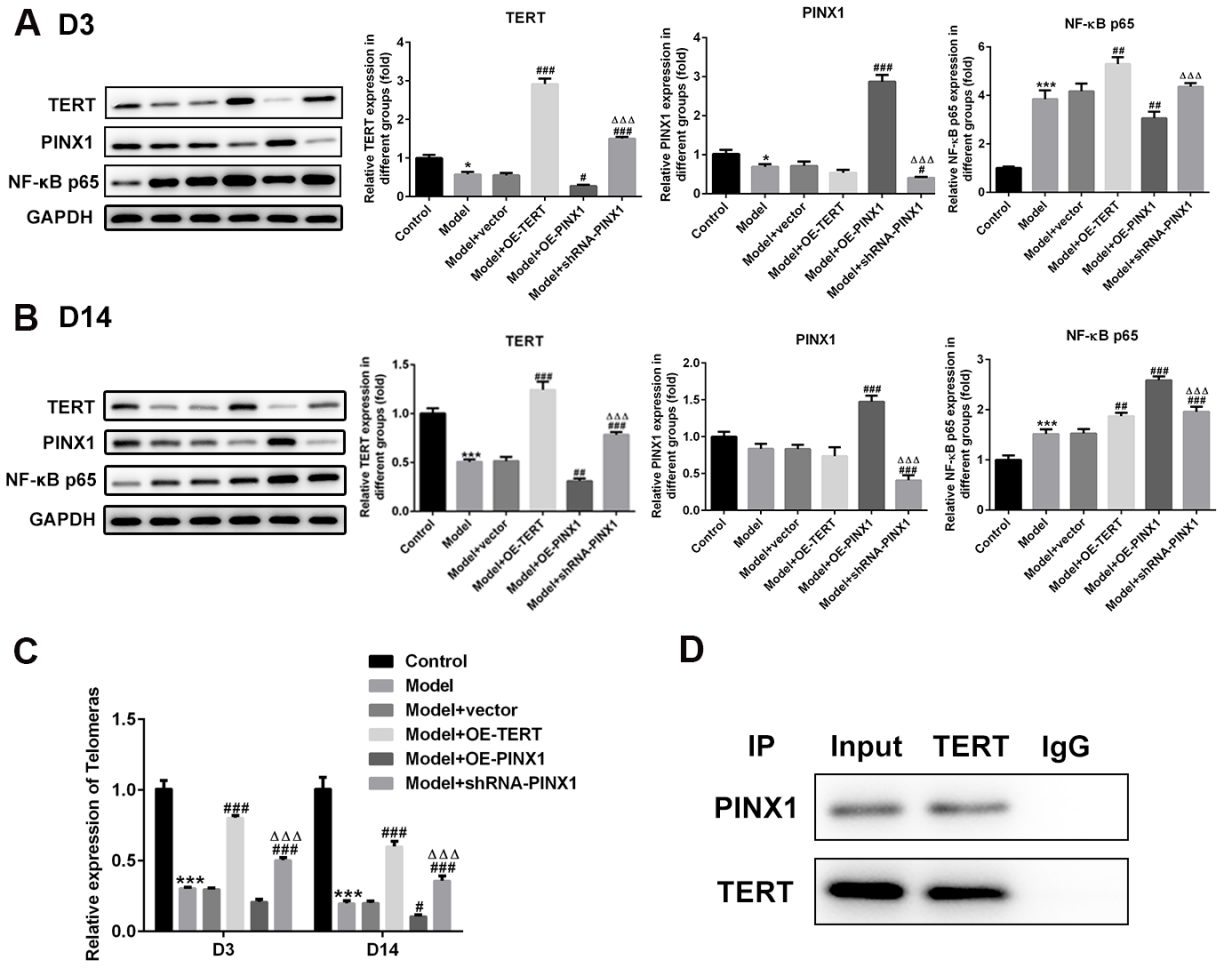
**PINX1 regulated NF-κB p65 expression and the telomerase activity via directly interacting with TERT.** The expression of TERT, PINX1, and NF-κB p65 on (**A**) D3 and (**B**) D14 was determined using western blot analysis. (**C**) The telomerase activity was measured using commercial kit. ^**^P<0.01, ^***^P<0.001 vs. Control; ^#^P<0.05, ^##^P<0.01, ^###^P<0.001 vs. Model+vector; ^ΔΔΔ^P<0.001 vs. Model+OE-PINX1. (**D**) Co-immunoprecipitation assay was carried out to verify the interactions between PINX1 and TERT.

To clarify the interactions between PINX1 and TERT, the levels of both of them and NF-κB p65 on D3 and D14 were detected using western blot analysis. As shown in Figure 6A, significantly reduced TERT and PINX1 expression and elevated NF-κB p65 expression were observed in the model group relative to the control group on D3. Overexpression of PINX1 dramatically inhibited the level of TERT, whereas PINX1 knockdown backfired. Remarkably, TERT overexpression or PINX1 silencing promoted the expression of NF-κB p65 (Figure 6A). On D14, obviously decreased level of NF-κB p65 was noticed when TERT overexpression or PINX1 silencing. However, PINX1-uregulation exhibited the highest expression of NF-κB p65 in lung tissues of LPS-induced lung injury rats (Figure 6B). Moreover, the telomerase activity was measured using commercial kits. Results of Figure 6C revealed that the activity of telomerase was reduced significantly in the model group on D3 and D14, and TERT overexpression or PINX1 silencing enhanced the activity of it while PINX1 overexpression showed the lowest level of telomerase activity. The STRING website (https://string-db.org/) predicted that TERT can interact with PINX1. Co-IP assay was carried out to verify the interaction. From the results in Figure 6D, we found that there was a strong interaction between TERT and PINX1. Overall, these data suggested that PINX1 regulated the telomerase activity via directly interacting with TERT.

## DISCUSSION

Sepsis is considered as a leading cause of mortality in intensive care unit patients, which can result in multiple organ failure, and lung serves as the most susceptible target organ in sepsis [14, 15]. In our current study, LPS was applied to establish the ALI rat model to explore the regulatory interactions between PINX1 and TERT from the acute stage to the convalescent phase of ALI rats. It was demonstrated that regulation of PINX1 expression could ameliorate lung injury and alleviate cell senescence during the convalescent phase by affecting the telomerase activity.

A growing body of evidence suggests that the potential mechanisms of ALI pathogenesis include inflammatory responses and apoptosis [16, 17]. It is considered that the essence of ALI is an excessive and uncontrolled inflammatory response. Extensive lung inflammation contributes to the destruction of the basement membrane and increased the permeability of the alveolar-capillary membrane [18]. Numerous studies unveil that an exaggerated inflammatory response can cause apoptosis, which plays a vital role in the pathogenesis of ALI [19, 20]. Accumulation of a mass of inflammatory factors in lung tissues has been demonstrated to lead to pulmonary cell apoptosis [21]. In addition, accumulation of apoptotic cells can enhance the levels of inflammatory factors [22]. Herein, inhibition of inflammation and apoptosis may protect cells against ALI. PINX1, an inhibitor of telomerase activity, is necessary for TERT elements to bind at telomeres and non-telomere sites [12]. And PINX1 is considered as a key component of TERT/telomerase homeostasis through its ability to bind to TERT and inhibit TERT activity [12]. It’s widely accepted that a telomere is a region of tandem repeats of short DNA sequences at the ends of chromosomes, which are crucial for its stability and the complete replication of the ends [23, 24]. Telomerases can effectively stabilize telomere length and thus stabilize cell reproduction. Therefore, cell senescence and proliferation depend on telomere stabilization and telomerase activation, which is of great significance for the normal cellular function [25]. It has been reported that inflammation is one of the influencing factors for cell senescence, among which the growth rate of telomere length shortening is considered to be one of the important factors [26]. Emerging evidence supports that TERT could attenuate lung fibrosis via protecting alveolar epithelial cells against senescence [13]. However, it is noteworthy that inhibition of TERT decreases the content of TNF-α by inactivation of NF-κB signaling PINX [12]. Further studies have found that PINX1 and p65 have a co-expression relationship, and the biphasic action of PINX1 is related to the different binding domains and corresponding functions of its C-terminal and N- terminal [12]. The present study revealed that TERT maintains cell stability during the acute phase (D3) of sepsis induced by LPS, but it also stimulates inflammation by stimulating NF-κB. Simultaneously, TERT is consumed during the inflammatory process. In the convalescent phase (D14), TERT plays a role in maintaining cell proliferation, reducing cell death, and maintaining cell stability. However, due to the depletion of TERT, the level of cell proliferation and apoptosis is still quite different from that of normal cells. Although PINX1 was also depleted in the acute phase, the relative ratio of PINX1 to TERT was changed, which was manifested in the remission phase as PINX1 inhibited TERT activity to a larger extent, resulting in irreparable telomere damage, cell damage and senescence.

Additionally, results in the present study suggested that overexpression of TERT led to increased inflammation in the acute phase of sepsis induced by LPS, and higher TERT level at convalescent phase promoted cell proliferation and reduced abnormal cell apoptosis, thus increasing the recovery rate. By contrast, PINX1 overexpression resulted in a reduced inflammatory response during the acute phase by inhibiting TERT and inhibition of NF-κB, but a heavier loss of TERT led to a slower recovery rate during the recovery phase. Moreover, inhibition of PINX1 also increased inflammation in the acute phase (TERT lost its original inhibition by PINX1), and recovery was accelerated appropriately in the convalescent phase, but the degree of recovery was between the model group and the TERT overexpression group.

In conclusion, the present study is the first to document the pivotal roles of PINX1 in the recovery of ALI. We demonstrated that regulation of PINX1 expression could ameliorate lung injury and alleviate cell senescence during the convalescent phase through affecting the telomerase activity. Our findings corroborated that PINX1 may serve as a potential therapeutic target in the treatment of ALI, which provides an innovative perspective for the clinical therapy for ALI. However, the lack of experiments *in vitro* is a limitation of the present research and therefore, a comprehensive analysis is required in the future.

## MATERIALS AND METHODS

### Animals

A total of 72 adult Sprague Dawley rats (200–250 g) obtained from Shanghai SLAC Laboratory Animal Company Ltd. (Shanghai, China) were used for the experiments. The rats were housed in a suitable environment at 21±3° C under a 12-h light–dark cycle. All animals were freely provided with normal diet and water. All animal procedures were approved by the Ethics Committee of the Qingdao Hospital of Traditional Chinese Medicine (Hiser Hospital).

### Groups

They were assigned on random into six groups (n = 12 in each group) according to the sacrifice time and treatments: (1) control group (rats with intratracheal instillation of normal saline); (2) model group (rats with intratracheal instillation of LPS); (3) model+vector group (rats exposed to LPS and injected with Ad-GFP vector via tail vein); (4) model+OE-TERT (rats exposed to LPS and injected with Ad-TERT via tail vein); (5) model+OE-PINX1 group (rats exposed to LPS and injected with Ad-PINX1 via tail vein); (6) model+shRNA-PINX1 group (rats exposed to LPS and injected with shRNA-PINX1 via tail vein). The tail vein injection of vector carrying TERT, PINX1 and shRNA-PINX1 were given 1 day, 7 days, and 14 days after the beginning of the experiment.

### Establishment of ALI rat model

The ALI rat model was established according to the previous studies [27, 28]. In brief, animals were anesthetized by intraperitoneal injection with 3% sodium pentobarbital. Then, 5 mg/kg LPS (Sigma Chemical Co., St. Louis, MO, USA) solution was used on rats to induce lung injury by intratracheal instillation. Rats in the control group were exposed to an equal volume of normal saline instead of LPS. All animals were humanely sacrificed on day 3 (D3) and day 14 (D14) after LPS stimulation as previous studies described [29, 30]. The blood samples and lung tissues were obtained to conduct the following experiments.

### Lung histological examination

The pathological changes of lung injury in rats on day 3 and day 14 after LPS stimulation were evaluated using hematoxylin and eosin (H&E) staining. The appropriate left lung was obtained and fixed with 10% formalin for 24 h, followed by the dehydration with ethanol and xylene and the embedding in paraffin wax. Subsequently, paraffin-embedded lung tissue was cut into 4 μm thick sections on glass slides. The sections were mounted on slides and stained with hematoxylin and eosin, followed by dehydration with graded ethanol and xylene. Images were photographed using a light microscope (Olympus Corp., Tokyo, Japan). The degree of pathological injury was scored based on edema, neutrophil infiltration, hemorrhage, and disorganization of lung parenchyma, as a previously scoring system described [31]. Higher scores indicate more severe lung damage.

### Collection of bronchoalveolar lavage fluid (BALF)

After the chest was opened, 1 mL physiological saline was used to lavage from the bronchus alveolar for three times and the interval was 1 minute each time. The total cell number in BALF was counted on the microscopic counting plate. Afterwards, BALF was centrifuged at 3000 rpm for 10 minutes to obtain the supernatant which was stored at −80° C until use.

### Test for the content of total proteins

A bicinchoninic acid (BCA) Protein Assay Kit (Beyotime, Shanghai) was employed to determine the protein concentration in BALF in accordance with the manufacturer’s guidelines. And the contents of cells in BALF were counted by an automatic cell counter.

### Measurement of inflammatory factors

The levels of inflammatory factors including TNF-α, IL-1β, IL-6, and IL-18 in BALF were determined using enzyme-linked immunosorbent assay (ELISA) kits, which were purchased from Shanghai Xitang Biotechnology Co., Ltd. (Shanghai, China). All the operations were conducted under the manufacturer’s guidelines. The experimental detection wavelength was 450 nm.

### Terminal-deoxynucleotidyl transferase mediated nick end labeling (TUNEL) assay

The apoptosis of lung tissues was assessed using a TUNEL apoptosis kit (Roch Applied Science, China). Briefly, lung tissues were washed with phosphate-buffered saline (PBS) and then fixed with 4% paraformaldehyde. The apoptotic cells in 4 μm thick section were visualized with the TUNEL staining according to the manufacturer’s instructions.

### Co-immunoprecipitation (IP) assay

For co-immunoprecipitation assays, cells were lysed in Lysis Buffer for IP (Beyotime Institute of Biotechnology). Lysates were incubated with indicated antibodies plus Protein A/G beads (Santa Cruz Biotechnology, Inc.). After washing the beads, immunoprecipitates were analyzed using western blot analysis.

### Reverse transcription-quantitative PCR (RT-qPCR)

Total RNAs in lung tissues were collected using TRIzol reagent (Invitrogen). A PrimeScript® RT reagent kit (Takara Bio, Inc.) was applied to synthesize the complementary DNA following the manufacturer’s recommendations. Subsequently, qPCR was carried out on an ABI PRISM 7500 Sequence Detector System (Applied Biosystems) using gene-specific primers for TERT and PINX1. Data were normalized to GAPDH. Relative mRNA expression was determined using the 2^-ΔΔCq^ method.

### Western blot analysis

Total proteins in lung tissues were extracted using lysis buffer containing a protease inhibitor cocktail (Sigma-Aldrich, MO, USA). A BCA Protein Assay Kit (Beyotime, Shanghai) was detected for protein concentration. Equal amount of proteins was resolved by 10% SDS-PAGE gel, followed by the electronic transfer onto PVDF membranes. Afterwards, the membranes were blocked utilizing 5% non-fat milk. Then, the membrane was incubated with primary antibodies at 4° C overnight. After rinsing with TBST for three times, these blots were incubated with horseradish peroxidase (HRP)-conjugated secondary antibody (Cell Signaling Technology, Inc., Boston, MA, USA). The blots were visualized using the Odyssey Infrared Imaging System (LI-COR Biosciences). The levels of protein expression were quantified using Image J software (National Institutes of Health). GAPDH was performed to normalize the loading. Anti-Bax (cat. no. 14796S), anti-cleaved caspase-3 (cat. no. 9664T), anti-NF-κB p65 (cat. no. 8242T) and anti-GAPDH (cat. no. 5174T) antibodies were obtained from Cell Signaling Technology, Inc. (Boston, MA, USA). Anti-Bcl-2 (cat. no. sc-7382), anti-TERT (cat. no. sc-377511) and anti-PINX1 (cat. no. sc-374113) antibodies were purchased from Santa Cruz Biotechnology, Inc. (Dallas, TX, USA).

### Statistical analysis

All of the data in the present study were analyzed by GraphPad Prism software, version 6.0 (GraphPad Software Inc., USA). The data are described as the mean ± standard deviation, which were performed three independent experiments. The statistical differences were calculated with unpaired student’s t-test or one way analysis of variance (ANOVA) followed by Tukey’s test. The P value of less than 0.05 was considered to be a significant difference.

### Ethics approval and consent to participate

All experimental protocols were approved by the Ethics Committee of the Qingdao Hospital of Traditional Chinese Medicine (Hiser hospital).
